# Supplementary figures and images for: Using multi-scale distribution and movement effects along a montane highway to identify optimal crossing locations for a large-bodied mammal community
Source: PeerJ. 2013 Oct 24;1:e189. doi: 10.7717/peerj.189 (PMC3817594; doi:10.7717/peerj.189)

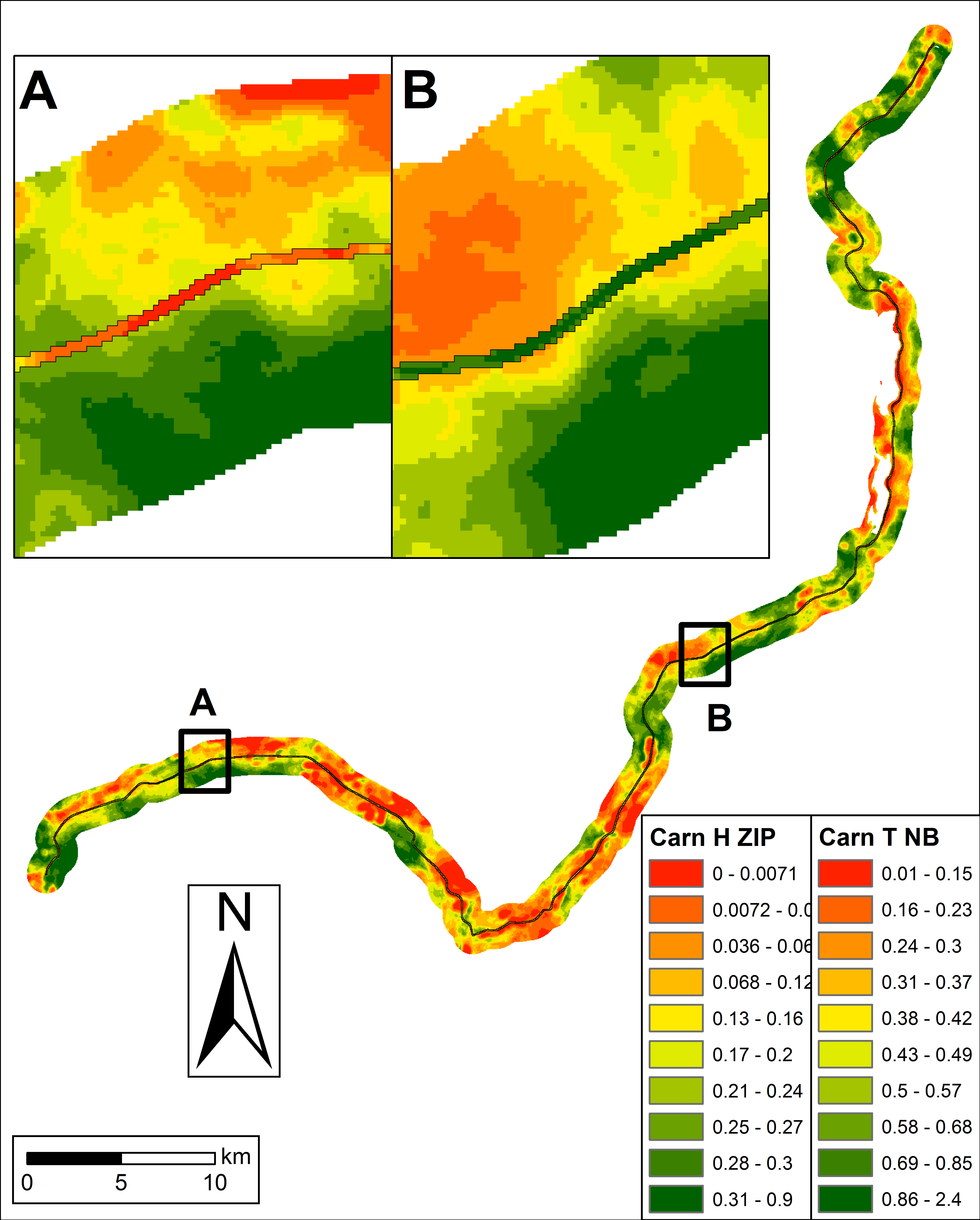

Supplement: Figure S1 [file peerj-01-189-s001.png]

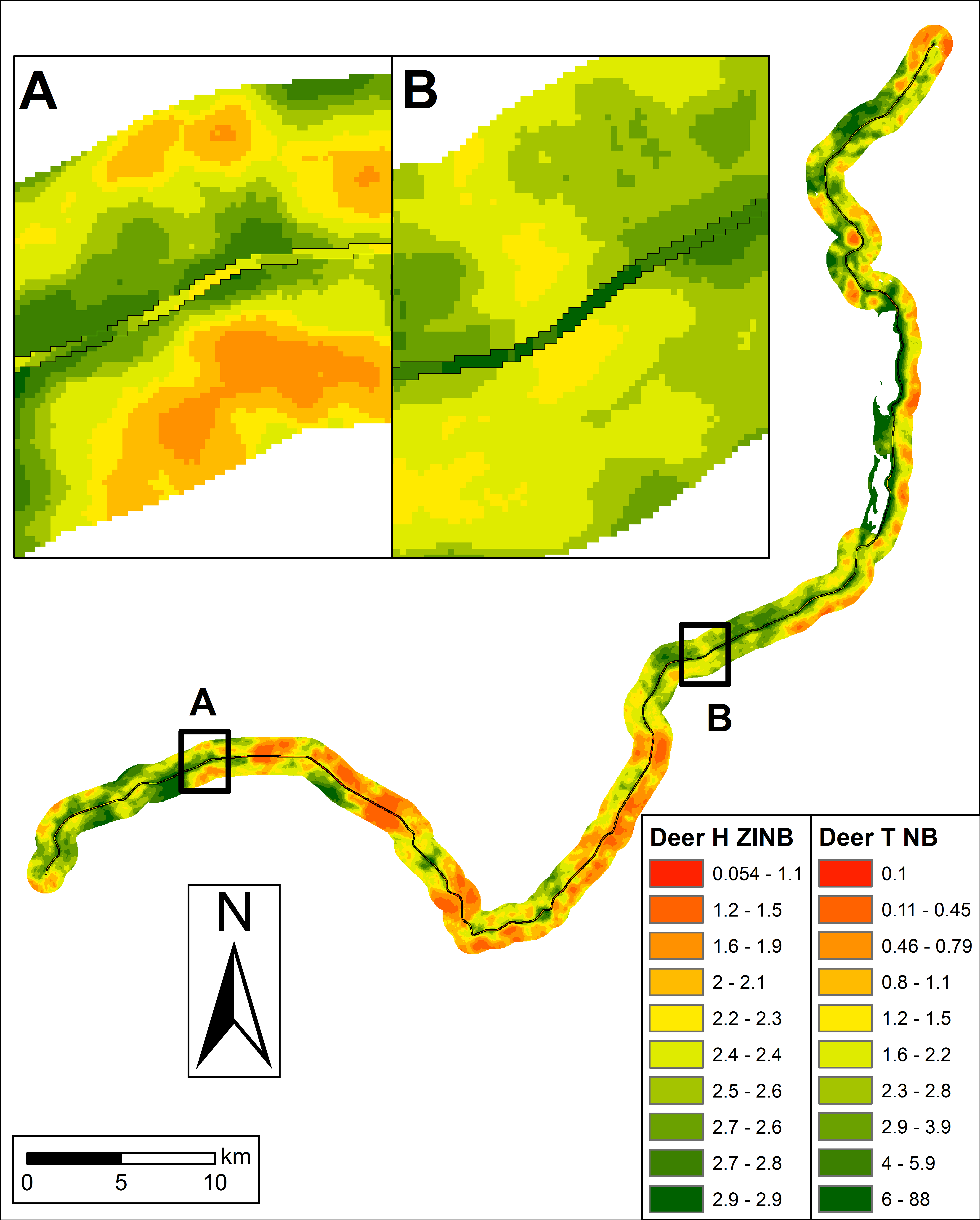

Supplement: Figure S2 [file peerj-01-189-s002.png]

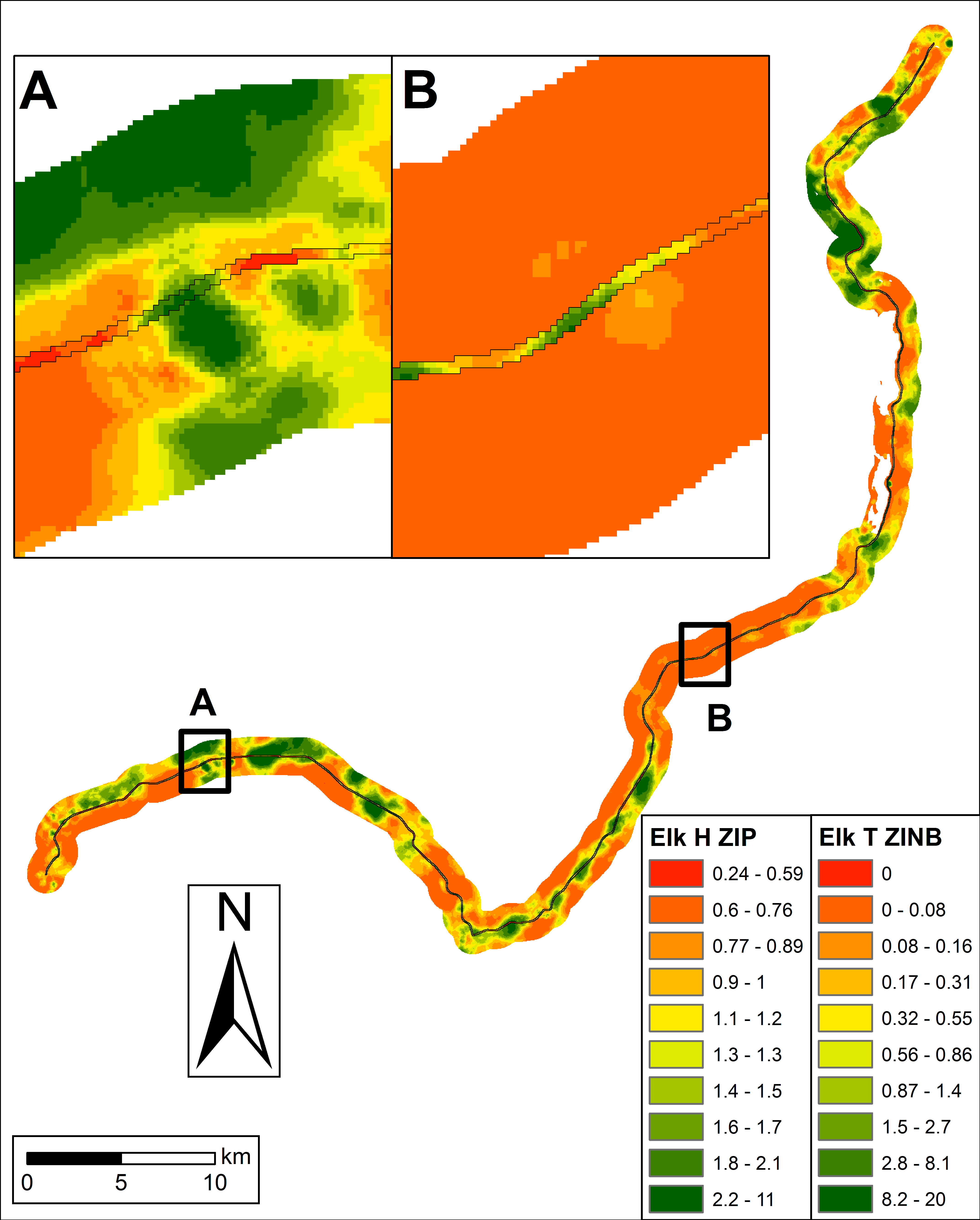

Supplement: Figure S3 [file peerj-01-189-s003.png]

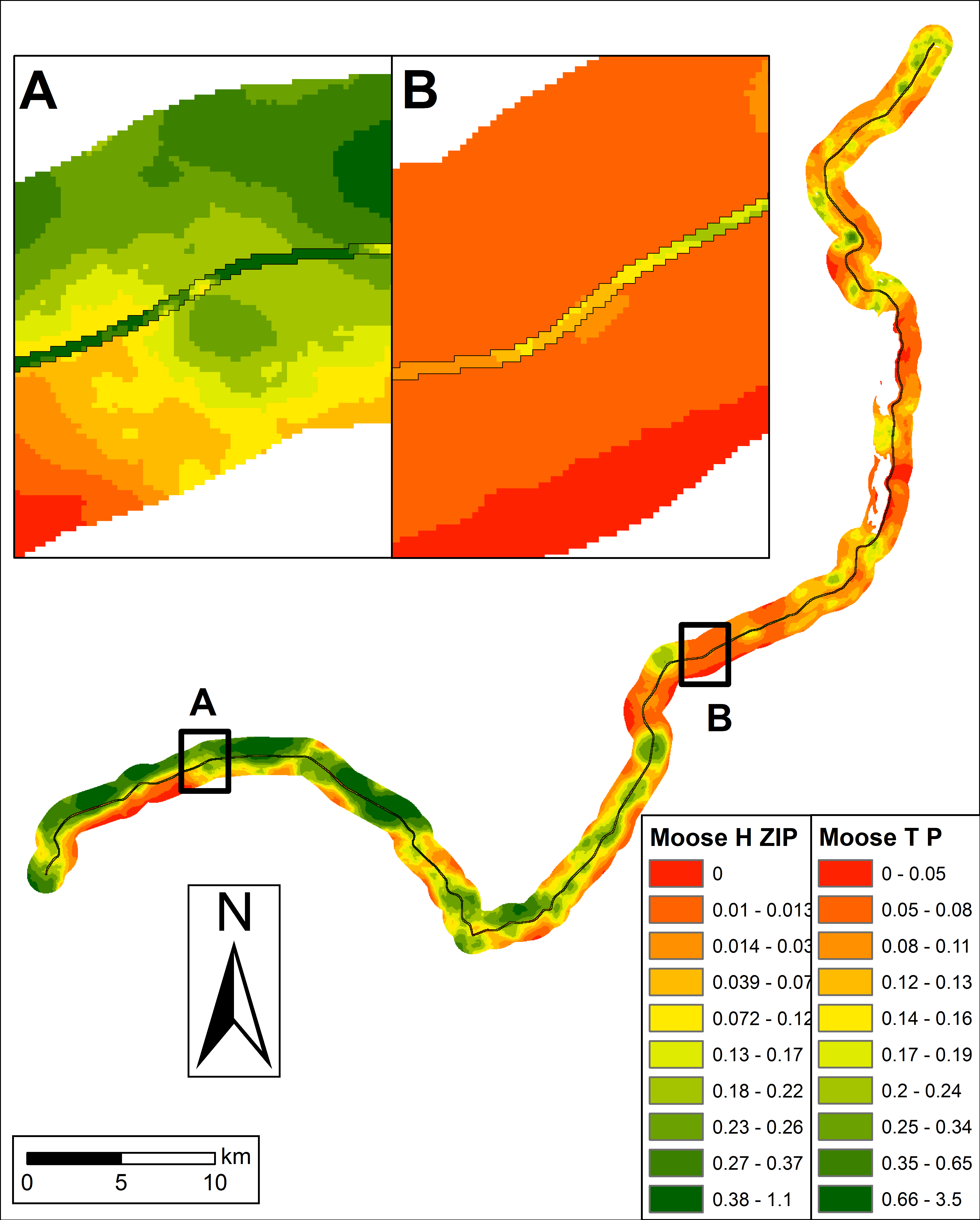

Supplement: Figure S4 [file peerj-01-189-s004.png]
